# Electroantennographic and Behavioral Responses of the Melon fly, *Zeugodacus cucurbitae* (Coquillett), to Volatile Compounds of Ridge Gourd, *Luffa acutangular* L.

**DOI:** 10.1007/s10886-024-01474-1

**Published:** 2024-02-19

**Authors:** Jing jing Wang, Chao Ma, Zhen ya Tian, Yong ping Zhou, Jin fang Yang, Xuyuan Gao, Hong song Chen, Wei hua Ma, Zhong shi Zhou

**Affiliations:** 1 State Key Laboratory for Biology of Plant Diseases and Insect Pests, Institute of Plant Protection, Chinese Academy of Agricultural Sciences, Beijing, 100193 China; 2 National Nanfan Research Institute, Chinese Academy of Agricultural Sciences, Sanya, 572019 China; 3 Guangxi Key Laboratory for Biology of Crop Diseases and Insect Pests, Institute of Plant Protection, Guangxi Academy of Agricultural Sciences, Nanning, 530007 China; 4 Hubei Insect Resources Utilization and Sustainable Pest Management Key Laboratory, College of Plant Science and Technology, Huazhong Agricultural University, Wuhan, 430070 China

**Keywords:** *Zeugodacus cucurbitae*, GC–MS, Plant Volatiles, EAG, Behavioral Responses

## Abstract

The melon fly, *Zeugodacus cucurbitae* (Coquillett)*,* is a major invasive pest, widely distributed in the Asia–Pacific region and some parts of Africa. Melon fly attractants could improve the effectiveness of current pest management measures. Previous studies have shown that some host fruits are attractive to melon flies but few have investigated the chemical compounds responsible for their attraction. In this study, we aimed to identify the volatile compounds from *Luffa acutangula* L that attract *Z. cucurbitae*. In headspace trapping, chemical profiling identified 19 compounds from ridge gourds, with 1-pentadecene being the major component. EAG results revealed that seven compounds elicited antennal responses in *Z. cucurbitae*, and significant differences in antennal responses between male and female *Z. cucurbitae* adults were recorded to *p*-xylene, alpha-pinene, and 1-octadecene. Behavioral experiments demonstrated that the EAG-active compounds methyl isovalerate and methyl myristate had either attractive or repellent effects on *Z. cucurbitae* at different concentrations, and 1-octadecene attracted *Z. cucurbitae.* Our findings provide a theoretical basis producing repellents or attractants for effective Integrated Pest Management of *Z. cucurbitae.*

**Supplementary Information:**

The online version contains supplementary material available at 10.1007/s10886-024-01474-1.

## Introduction

The melon fly, *Zeugodacus cucurbitae* (Coquillett) (Diptera: Tephritidae) is native to India and widely distributed in tropical and subtropical regions worldwide (Dhillon et al. 2005; Mir et al. 2017). The female melon flies lay eggs in fruits, and the larvae largely feed on fruits causing stunted development and rotting of infested fruits, resulting in 30–100% loss (Christenson and Foote 1960; Dhillon et al. 2005). Currently, melon fly infestations are primarily controlled using insecticides. However, this is not a suitable control method owing to high levels of insecticide resistance and pesticide residues, which have harmful effects on human health and the environment (Aktar et al. 2009; Cha et al. 2023; Curl et al. 2020; Hsu et al. 2021;). Moreover, the control of melon flies is mainly aimed at male insects, as in male annihilation and mass trapping to attract and kill male flies (Jang et al. 2017a, b; Lehman et al. 2019; Stringer et al. 2019;). However, females lay eggs on the surface of fruits which is the main cause of harm, and it may be possible to identify potential ovipositional attractants and kairomones that are effective in trapping female melon flies, leading to better control. The chemicals that attract females are food-bait attractants, such as fermented sugars, hydrolyzed proteins, and yeast. Unfortunately, these baits are usually liquid, may lack potency, have limited field life, and often attract non-target species, so non-food female attractants are needed. (Barry et al. 2006; Dhillon et al. 2005; Vargas et al. 2015, 2018).

Most plants have volatile chemical signals that affect insect behavior, such as locating hosts, oviposition sites, mates, and avoiding predators (Hansson and Stensmyr 2011; Leal 2013; Lin et al. 2015; Suh et al. 2014; Wang et al. 2016), which can play important roles in plant–insect interactions (Germain et al. 2010; Nascimento et al. 2021; Song et al. 2020). The most promising source of female attractants is the host plant, such as host fruits and leaves as females prefer fruit volatiles when searching for spawning sites (Siderhurst and Jang 2010). These behavior-altering host plant volatiles could be studied in the development of female fly attractants, providing a promising option for IPM of melon fly (Shivaramu et al. 2022). Female melon flies use host plant volatiles to find suitable hosts to oviposit, nd these volatile chemical cues are being explored as effective attractants in several Tephritidae. For example, 1-octen-3-ol, a volatile from mango, guides the oviposition behavior of *Bactrocera dorsalis* (Xu et al. 2023). Volatiles from *Prunus cerasus* are the main chemical signals used by *Rhagoletis cerasi* in seeking host plants (Būda et al. 2022). *Anastrepha fraterculus* males are sexually stimulated by the aroma of fruit of its native host *Psidium guajava* (guava) (Bachmann et al. 2023). Linalool acts as an oviposition deterrent on female *Ceratitis capitata* (Papanastasiou et al. 2020). Olive fruit volatiles may serve as kairomones for female *Bactrocera oleae* (Giunti et al. 2020), and *Anastrepha fraterculus* females show a preference for males that were exposed to guava volatiles (Bachmann et al. 2019).

While melon fly is a serious pest of many tropical crops, causing damage to at least 81 host species, Cucurbitaceae plants are the preferred hosts (Dhillon et al. 2005). Host plant volatiles are known to be used as cues by female *Z. cucurbitae* to locate potential hosts and have been studied as attractive sources for detection and control (Piñero et al. 2020, 2021; Vargas et al. 2018). Freshly sliced cucumbers are particularly attractive, and a mixture of cucumber volatiles containing nine compounds has been developed into a synthetic attractant (Siderhurst and Jang 2010). Previous studies have shown that several host fruits attract gravid female melon flies, However, the isolation and identification of attractants from these fruits is limited (Jang et al. 2017a, b; Piñero et al. 2021; Siderhurst and Jang 2010; Subhash et al. 2018;). The behavioral response of insect species to olfactory cues is not only affected by environmental conditions but also by age, life history, and other physiological states (Epsky et al. 2014; Piñero et al. 2021). Melon flies seek food sources during the pre-oviposition period and host fruits after mating. Previous studies have reported that ridge gourd volatiles are attractive to gravid female melon flies. However, their effect before mating remains unclear (Shivaramu et al. 2022).

Therefore, we investigated the attraction of immature male and female adult *Z. cucurbitae* to volatiles released from ridge gourds. Specifically, we used electrophysiological, chemical, and behavioral responses to identify the chemicals that mediate the interaction between melon flies and ridge gourds. The results of this study can provide reference for the development of attractants for effective control of melon flies.

## Materials and Methods

### Insects

Melon flies were obtained from rotten fruits in the fields around Guangxi and kept in insect rearing cages (30 cm × 30 cm × 30 cm) at ambient lab conditions (26 ± 1 ℃, 70 ± 10% relative humidity and 14:10 light: dark cycle) for adults to emerge and be maintained for over 20 generations. The emerged adults were fed water and a solid diet (1:2 yeast:sugar mixture), which were renewed every five days. Three- to seven-day-old unmated female and male adults were used for the electroantennography (EAG) and behavioral experiments. Before the EAG and behavioral experiments, the insects were starved for 6 h in the absence of food odors.

### Host Plants

Ridge gourds provided by the Institute of Vegetables, Guangxi Academy of Agricultural Sciences were planted in the Beaeful South Crop Integrated Cultivation Base of Nanning City, Guangxi Zhuang Autonomous Region, under conventional water and fertilizer management. Undamaged fruits at similar-ripeness stages were used for headspace volatile collections.

### Collection of Plant Volatiles

Dynamic headspace sampling (DHS) was used to collect volatiles from ridge gourds. Before use, the sampling cylinder (radius 20 cm, height 60 cm) gas-washing cylinder, silicone tube, and other instruments were cleaned with 75% absolute ethanol and heated at 180 °C for 6 h to eliminate odors. The adsorption column was filled with 200 mg PoraPak Q (80-100mesh, SigmaAldrich-ShangHai) adsorbent. The air sampler, carbon column, water column, sampling cylinder, and adsorption column weree connected with silicone tubing, and each connection was sealed with sealing film to avoid air leakage of the collection device. The air flow of the air sampler was adjusted to 4 L/min, and the collection was continued for 24 h and repeated 4 times. In order to avoid the influence of the sampling cylinder itself on the analysis results, a sample of the empty sampling cylinder was also collected as the experimental control. After the collection, volatiles were removed from the adsorption column with 500 ul of *n*-hexane. In order to get as many samples as possible, the five collected eluent were mixed into one sample, and the sample was concentrated to 0.5 ml under a gentle stream of nitrogen. The extracted samples were stored at -20℃ until use.

### Gas Chromatography-Mass Spectrometry (GC–MS)

The extracts were analyzed by Gas chromatography coupled with mass spectrometry (GC–MS) using an Agilent 7890N-5975C (Agilent Technologies-USA) fitted with a DB-5MS column (30 m × 250 μm × 0.25 μm, Agilent). Aliquots of 1 µl of the extracts were injected in splitless mode using helium as the carrier gas at a flow rate of 1 mL/min. The oven temperature was programmed as follows: 40 ℃ for 5 min, 4 ℃/min to 120 ℃ for 3 min, 10 ℃/min to 220℃ for 10 min, and 30 ℃/min to 280 ℃ for 6 min. Ionization was by electron impact at 70 eV with a scan range of m/z 31–450 at a source temperature of 250 °C. Compounds were preliminarily identified through the NIST2017 spectrum library through ChemStation, combined with the analysis of the automatic mass spectrometry deconvolution Qualitative System (AMDIS), and further confirmed by comparison of the retention indices (RI) relative to retention times of *n*-alkanes with those in the NIST library.

### Electroantennography (EAG)

Each volatile of interest was dissolved in *n*-hexane to prepare 0.01, 0.1, 1, 10, and 100 µg/µL solutions, and the solutions were stored at -20 ℃ until required; *n*-hexane was used as a negative control. All chemicals used in this study were purchased from Sigma-Aldrich.(ShangHai).

Dose–response experiments were carried out for individual volatiles by EAG (Syntech IDAC-2, The Netherlands). The head of *Z. cucurbitae* was excised with a blade, the tip of the antenna (1 mm) was removed, and the preparation was immediately attached to electrodes by gently inserting the tip of the antenna into the recording electrode and the base of the antenna into the ground electrode with forceps. The glass electrodes were filled with 0.1 mol/L KCl. using a forceps.

Each test solution (10 µL) was applied to filter paper strips (40 mm wide × 5 cm long) that served as the olfactory stimulus. The filter paper was placed into a 10-cm sample tube with the end connected to the odor stimulus control device (CS-55, Syntech, The Netherlands). Air (400 mL/min) filtered by charcoal was constantly directed onto the antennal portions through a stainless-steel delivery tube, and the outlet was approximately 1 cm away from the antenna. Test compounds were delivered to the antenna of mounted insects through 0.5 s air pulse stimulations. The interval between each stimulation was 30 s, which guaranteed the recovery of antennae receptors. The order of stimulation was *n*-hexane, then the tested volatiles, then *n*-hexane, and EAG responses to *n*-hexane before and after the test chemicals were used as controls. EAG amplitude values were averaged for the control before and after stimulation by each test compound. Relative EAG response values were calculated as (CT-CK)/CK, where CT is the EAG amplitude of tested stimulus and CK is the average EAG amplitude of the control. For each compound, five concentrations (from 0.01 to 100 µg/µL) were tested on at least fifteen different females and males, and each antenna was tested only once.

### Dual Choice Behavioral Assays

Trap assays were performed as previously described (Qiao et al. 2019). Melon flies (100 females and 100 males, 3–7 d old, starved for 6 h) were introduced into a test chamber, comprising a rearing cage (30 cm × 30 cm × 30 cm), and two 150 mL white transparent plastic cups (6.5 cm × 6.5 cm × 4 cm) with a cut pipette tip (tip opening, 0.5 cm) placed in opposite corners. One of the cups contained a absorbent cotton impregnated with 1 mL of *n*-hexane (control), whilst the other cup contained 1 mL of the odorants (prepared with *n*-hexane at five doses 0.01, 0.1, 1, 10, and 100 µg/µL). The numbers of *Z. cucurbitae* inside and outside the traps were counted after 24 h. The odorant trap was recorded as an attractive choice, whilst the n-hexane trap was recorded as avoidance. Each experiment was repeated at least five times, cleaning the cups with anhydrous ethanol between repetitions and swapping the positions of the two cups to avoid contamination and orientation cues. Experiments began at the same time each day and were carried out in a climate chamber programmed for a 14L:10D photoperiod, constant temperature of 26 ± 1 ℃, and 70 ± 10% relative humidity.

### Data Analysis

All statistical analyses were performed using IBM SPSS20. The EAG responses were analyzed using one-way ANOVA with Bonferroni’s multiple comparison tests and the comparison between male and female response was tested by Independent-Sample *t*-test. Trap responses were analyzed by χ^2^ (Chi-square) test. Differences were considered significant at *P* < 0.05. All illustrations were performed using Prism 8 (GraphPad Software).

## Results

### Chemical Analysis of *L. acutangula* Volatiles

The GC–MS results revealed that volatiles from *L. acutangular* contained 19 components, including six esters, six alkanes, three alkenes, two alcohols, one aldehyde, and one aromatic. Among them, 1-pentadecene was the major component at 11.18% (Table 1).

**Table 1:** Identification of volatile compounds from *Luffa acutangular*

No	Retention time (min)	RI^a^	RI^b^	Compound	Abundance (%)	CAS #
1	2.90	697	700	heptane	3.06	142–82-5
2	5.89	770	772	methyl isovalerate	3.60	556–24-1
3	7.47	863	865	p-xylene	5.84	106–42-3
4	10.08	927	937	alpha-pinene	4.80	80–56-8
5	28.46	1399	1400	tetradecane	5.40	629–59-4
6	30.88	1488	1492	1-pentadecene	11.18	13360–61-7
7	31.38	1498	1500	pentadecane	4.80	629–62-9
8	33.11	1588	1591	β-cyclocitral	7.36	432–25-7
9	33.30	1598	1600	hexadecane	4.26	544–76-3
10	34.52	1679	1676	1-tetradecanol	6.66	112–721
11	34.80	1698	1700	heptadecane	3.98	629–78-7
12	35.17	1726	1725	methyl myristate	7.69	124–10-7
13	36.21	1808	1795	phytane	4.75	638–36-8
14	37.04	1880	1880	1-hexadecanol	4.80	36653–82-4
15	37.53	1926	1926	methyl palmitate	3.11	112–39-0
16	38.29	1998	1993	ethyl palmitate	6.55	628–97-7
17	39.29	2082	2082	1-octadecene	3.00	112–88-9
18	39.87	2124	2128	methyl stearate	4.91	112–61-8
19	43.51	2325	2329	methyl arachidate	4.26	1120–28-1

^a^RI actual measurement value. ^b^RI database search value

### EAG Responses

**Fig. 1:**
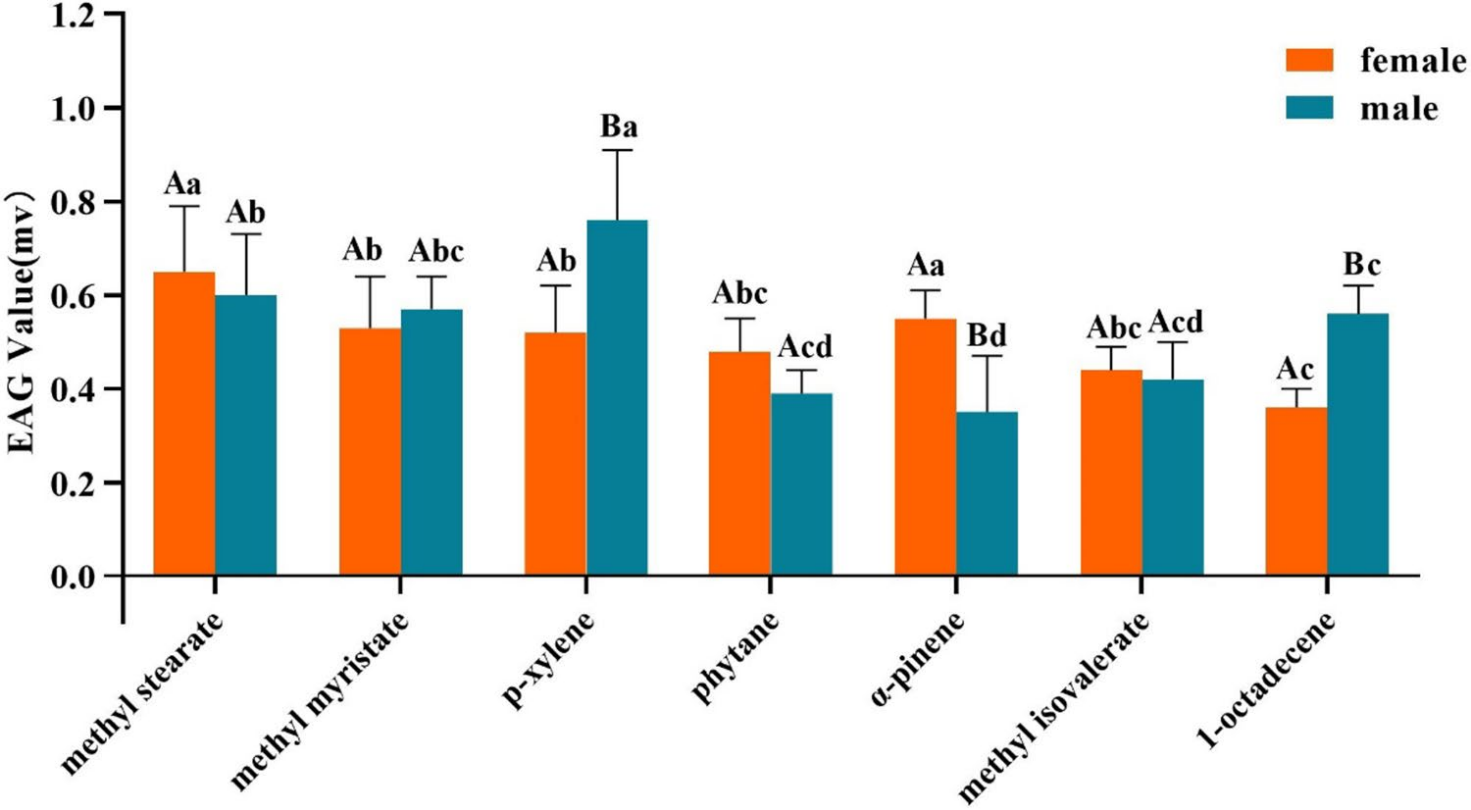
EAG response of *Zeugodacus cucurbitae* adult females and males to volatile compounds of *Luffa acutangular*. Significant differences between male and female are indicated by capital letter, and significant differences between different odors are indicated by lowercase letters (*P* < 0.05)

Of the 19 compounds detected in the headspace of *L. acutangula*, seven elicited antennal responses in EAG experiments, namely, methyl stearate, methyl myristate, p-xylene, phytane, α-pinene, methyl isovalerate, and 1-octadecene (Fig. 1). Among them, methyl stearate elicited high EAG responses and 1-octadecene elicited weak EAG responses from *Z. cucurbitae* adult males and females. In addition, the amplitude of EAG responses induced by *p*-xylene, α-pinene and 1-octadecene varied significantly between females and males. Statistical parameters are shown in the Supplementary Material Table S1.

**Fig. 2:**
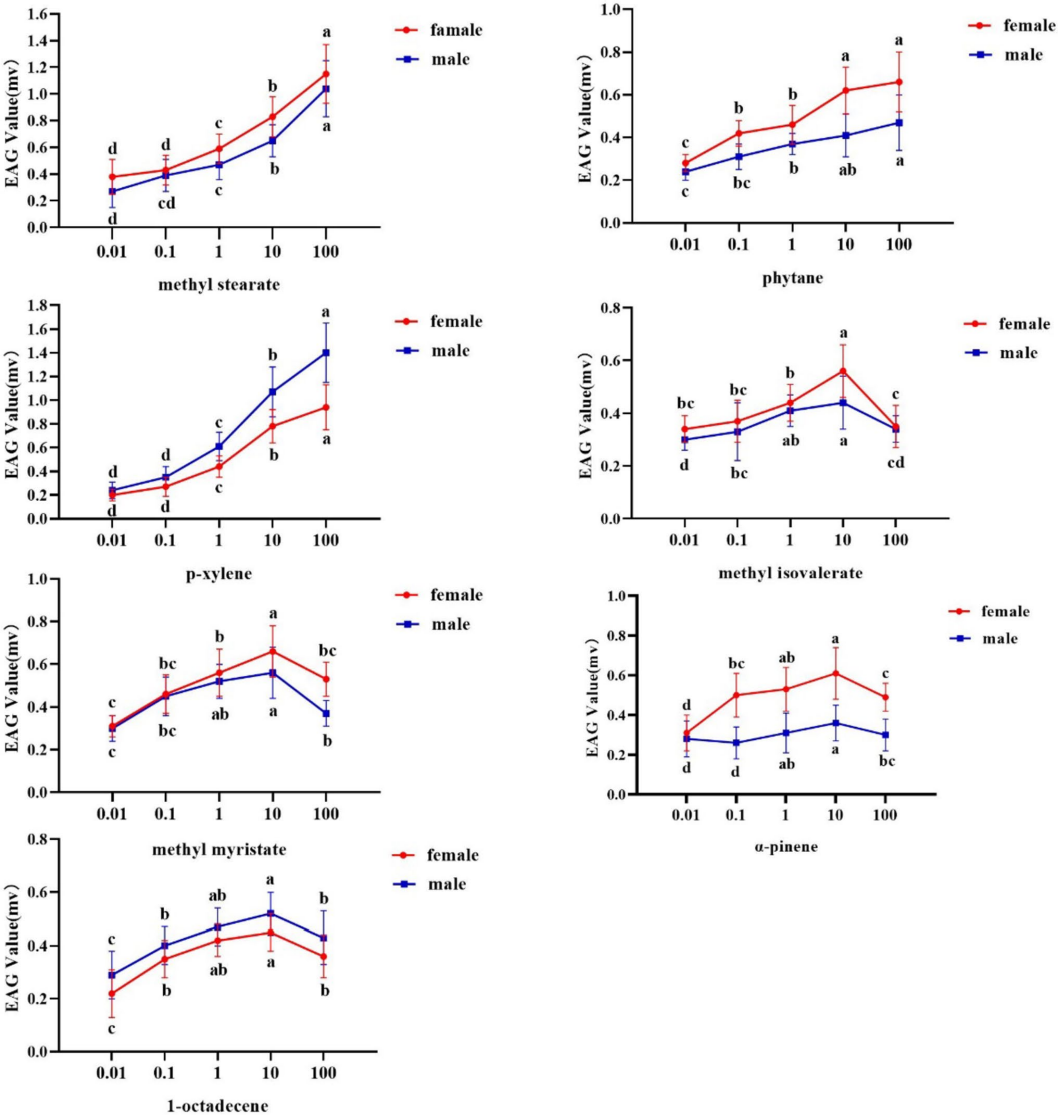
Dose–response of *Zeugodacus cucurbitae* adult females and males to volatiles of *Luffa. acutangular*. Significant differences among different doses are indicated by lowercase letters: the same letter means no difference, while different letters mean significant difference (*P* < 0.05)

The dose responses of EAG-active volatiles are shown in Fig. 2. For most test stimuli, the antennal responses of both sexes increased with compound concentration. However, there were different threshold values for the antennal responses of *Z. cucurbitae* to different volatiles. The antennal responses of *Z. cucurbitae* to methyl stearate, *p*-xylene, and phytane reached maxima at 100 µg/µL, to methyl myristate, α-pinene, methyl isovalerate, and at 10 µg/µL for 1-octadecene. Statistical paramters are shown in the Supplementary Material Table S2.

### Behavioral Responses

**Fig. 3:**
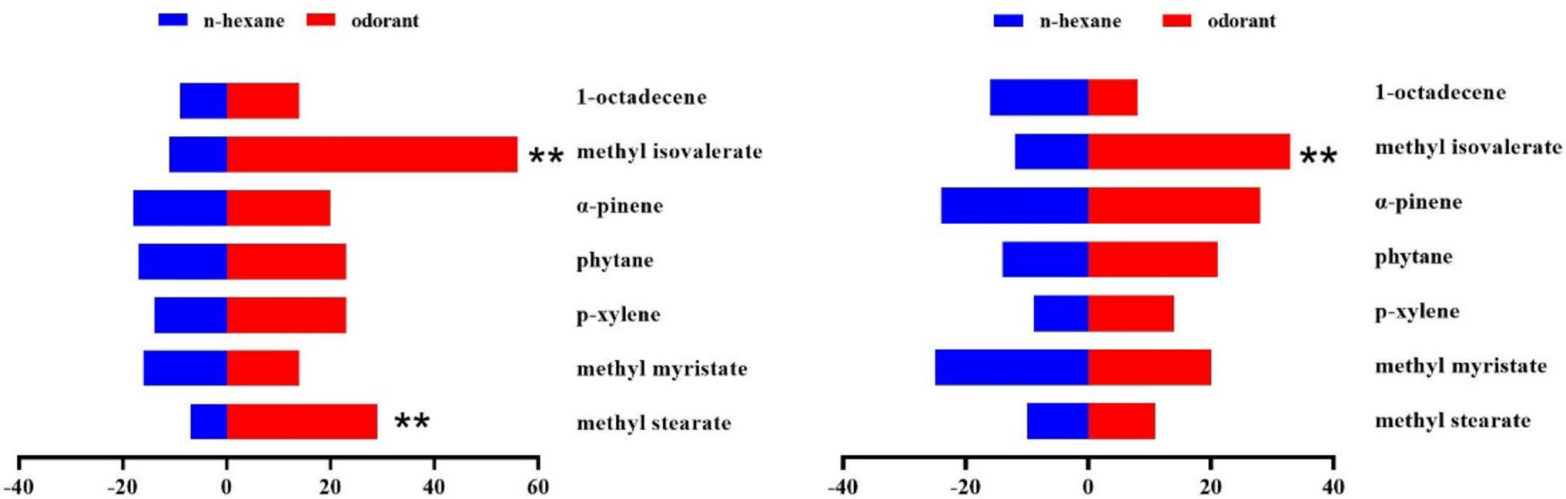
Behavioral responses of *Zeugodacus cucurbitae* adult females and males (*N* = 100) to 100 µg/µL *Luffa acutangular* EAG active compounds. A positive value of 0 on the right represents attractive effect, and a negative value on the left represents repellent effect. * indicates significant difference at the *P* < 0.05 and ** at the (*P* < 0.01) level in χ^2^ test between the treatment and the control

**Fig. 4:**
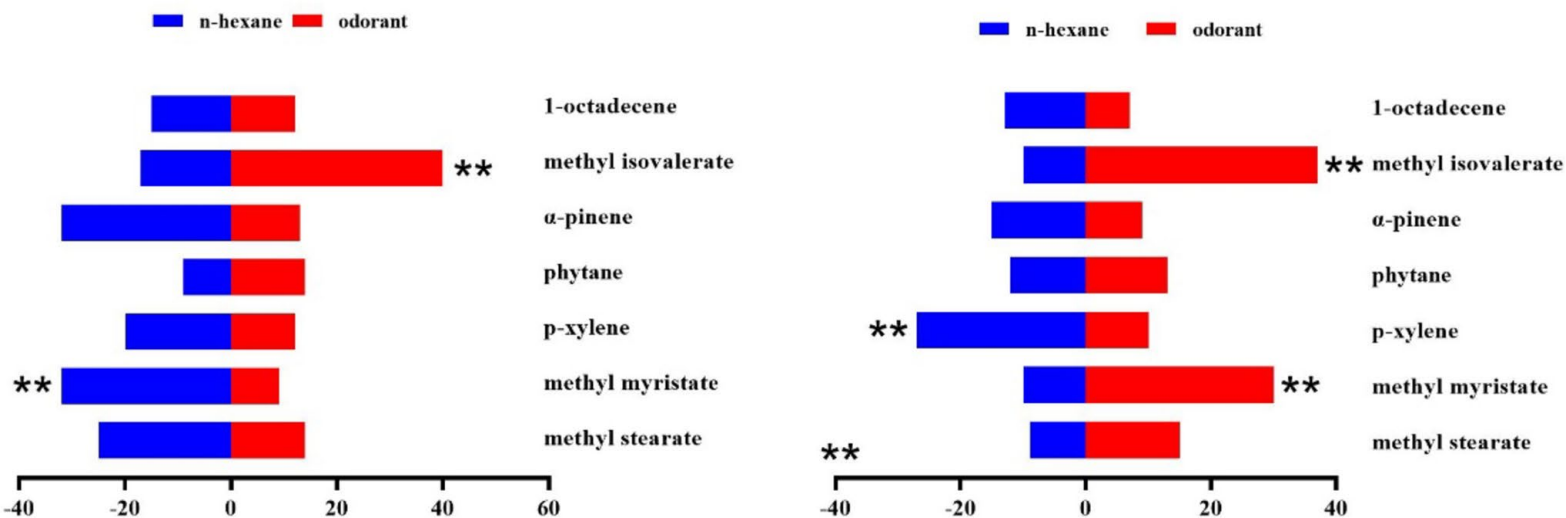
Behavioral responses of *Zeugodacus cucurbitae* adult females and males (*N* = 100) to 10 µg/µL *Luffa acutangular* EAG active compounds. A positive value of 0 on the right represents attractive effect, and a negative value on the left represents repellent effect. * indicates significant difference at the *P* < 0.05 and ** at the (*P* < 0.01) level in χ^2^ test between the treatment and the control

**Fig. 5:**
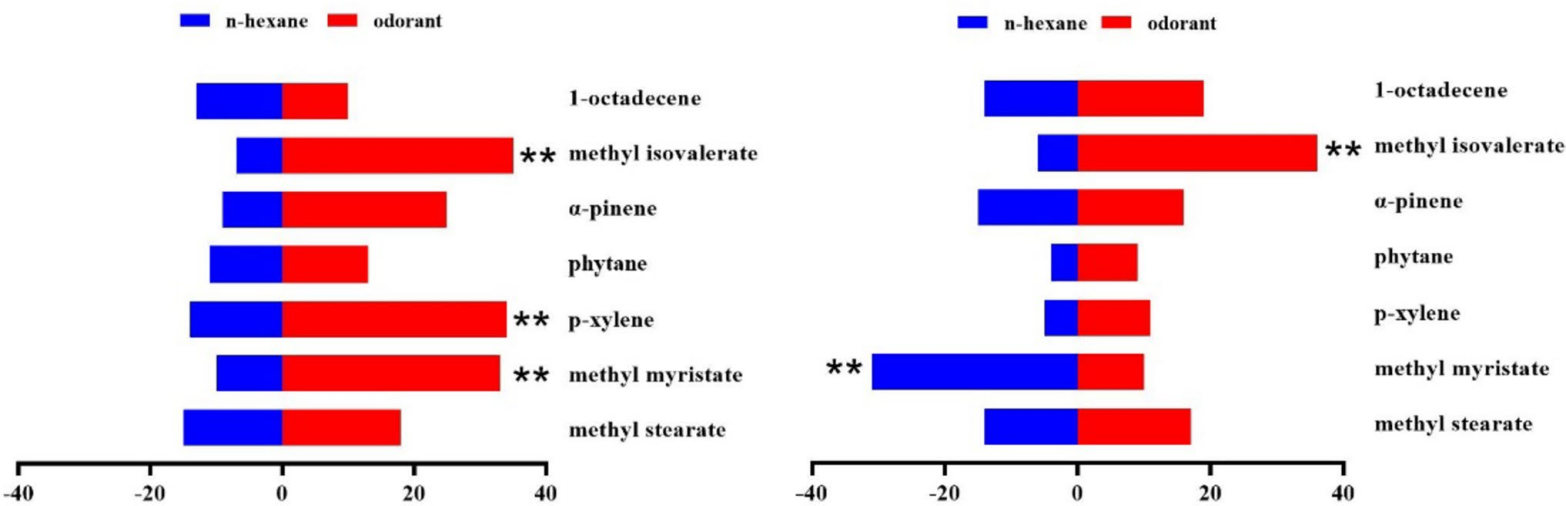
Behavioral responses of *Zeugodacus cucurbitae* adult females and males (*N* = 100) to 1 µg/µL *Luffa acutangular* EAG active compounds. A positive value of 0 on the right represents attractive effect, and a negative value on the left represents repellent effect. * indicates significant difference at the *P* < 0.05 and ** at the (*P* < 0.01) level in χ^2^ test between the treatment and the control

**Fig. 6:**
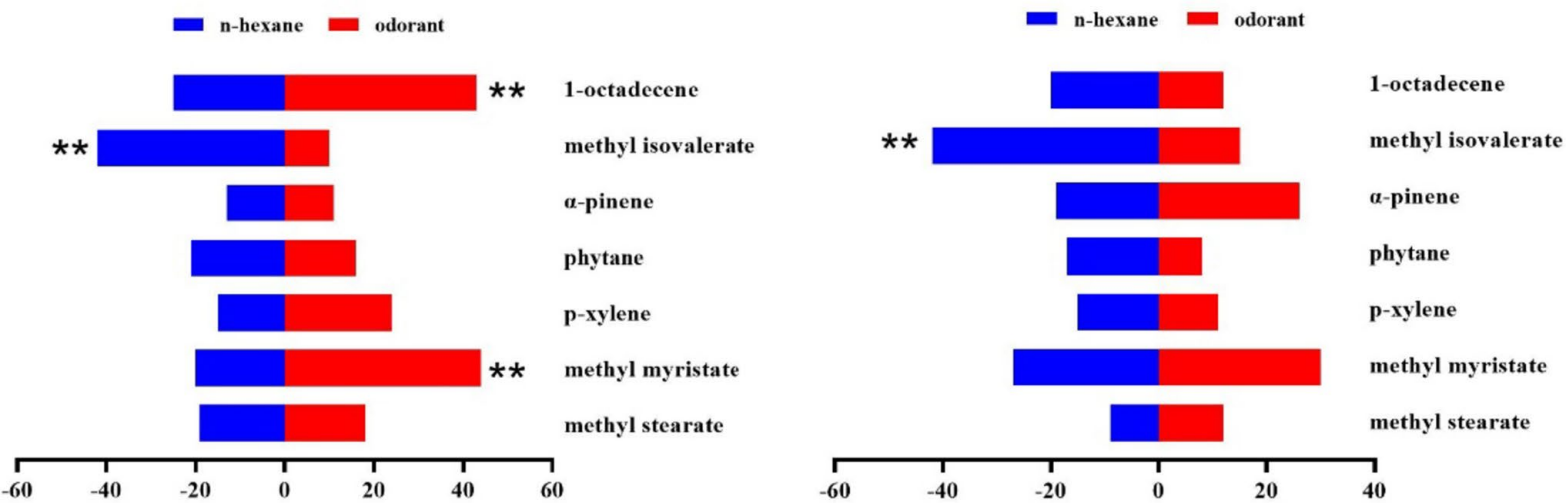
Behavioral responses of *Zeugodacus cucurbitae* adult females and males (*N* = 100) to 0.1 µg/µL *Luffa acutangular* EAG active compounds. A positive value of 0 on the right represents attractive effect, and a negative value on the left represents repellent effect. * indicates significant difference at the *P* < 0.05 and ** at the (*P* < 0.01) level in χ^2^ test between the treatment and the control

**Fig. 7:**
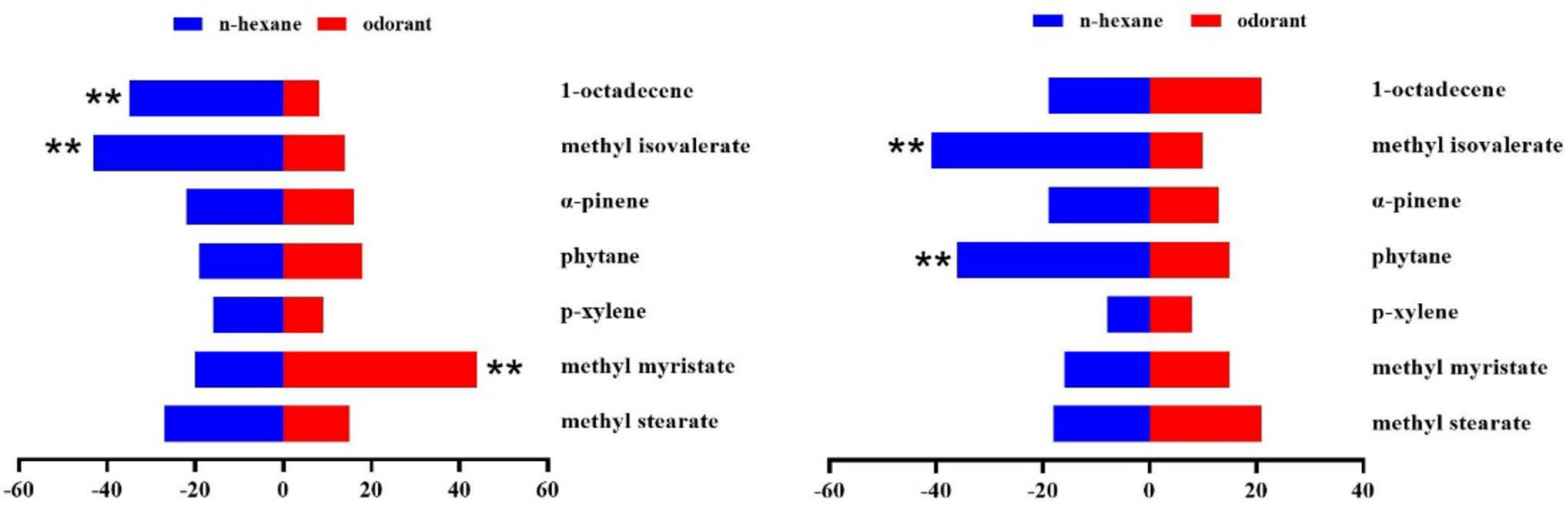
Behavioral responses of *Zeugodacus cucurbitae* adult females and males (*N* = 100) to 10 µg/µL *Luffa acutangular* EAG active compounds. A positive value of 0 on the right represents attractive effect, and a negative value on the left represents repellent effect. * indicates significant difference at the *P* < 0.05 and ** at the (*P* < 0.01) level in χ^2^ test between the treatment and the control

In the behavioral trapping experiment, melon flies showed significant sex- and concentration-dependent responses to the ten EAG-active compounds tested separately. At all test concentrations, methyl isovalerate was attractive to both sexes of *Z. cucurbitae* at 100, 10 and 1 µg/µL, but repellent to both sexes at 0.1 and 0.01 µg/µL (Figs. 3, 4, 5, 6, and 7). In addition, sex differences were apparent in the response of *Z. cucurbitae* to methyl myristate, which changed from repellent (10 μg/μL) to attractive (1–0.01 μg/μL) for females and from attractive (10 μg/μL) to repellent (1 μg/μL) for males (Figs. 4, 5, 6, and 7). Additionally, 1-octadecene changed from attractive (0.1 μg/μL) to repellent (0.01 μg/μL) in female (Figs. 6 and 7). Statistical data are shown in Supplementary Material Tables S4-S7.

## Discussion

Volatile chemical signals released by host plants are important for melon flies to find suitable hosts, feed, and oviposit. Melon flies prefer cucurbits as host plants, and their attraction to several fruits, including cucumbers, bitter gourds, snake gourds, and zucchini, has been documented (Piñero et al. 2021; Subhashet al. 2018). However, only cucumber volatiles have been studied as attractants of female *Z. cucurbitae*, and a nine-compound synthetic attractant was developed that gave captures of melon flies two-fold higher than with Solulys protein bait (Siderhurst and Jang 2010). Therefore, the search for attractants from other hosts is important.

In our study, using EAG, GC–MS analysis and dual choice behavioral assays, we identified compounds from ridge gourd that were both electrophysiologically and behaviorally active. The active compounds identified belong to the chemical classes alkenes, esters, aldehydes, alcohols, alkanes, and aromatics commonly produced by the fruits and flowers of various plants. In the current study, antennal responses to seven EAG compounds were detected, including methyl stearate, methyl myristate, *p*-xylene, phytane, α-pinene, methyl isovalerate and 1-octadecene. Thus far, compounds emitted by ridge gourd have been poorly studied and six EAG-active compounds were identified as α-pinene, 1-octen-3-ol, p-cymene, p-ethyl-benzaldehyde, methyl salicylate and *p*-cymen-7-ol (Shivaramu et al. 2022). The low similarity with our results could be due to both methodological differences and a significant difference in temperature systems used for the collection of compounds and ridge gourd varieties (Shivaramu et al. 2022).

In behavioral bioassays, *Z. cucurbitae* showed varying behavioral responses to individual compounds. Methyl isovalerate, methyl myristate, and 1-octadecene were found to have both attractive and repellent effects on *Z. cucurbitae*. In addition to causing responses in melon flies, these volatiles can also stimulate responses in other species. The effect of methyl isovalerate on melon flies changed from attractive to repellent. According to previous reports, methyl isovalerate in *Fusarium solani* attracts *Xylosandrus compactus* (Egonyu and Torto 2018), and repels flies (Henderson et al. 1991). 1-octadecene, which attracted and repelled melon fly in our study, is a repellent component of *Spodoptera frugiperda* (Kong et al. 2023)*.* Previous studies have shown that methyl myristate is attractive to *Varroa destructor* (Liu et al. 2022), *Drosophila melanogaster* (Dweck et al. 2015; Keesey et al. 2017)*,* and *Drosophila suzukii* (Matsumura) (Tait et al. 2020) but repellent to *Psoroptes ovis* (Hering) (Dunn et al. 2019). The combination of volatiles, as opposed to a single active substance, may be the reason for the differing results in behavioral responses (Baig et al. 2023; Stübner and Steinhaus 2023).

More often, herbivorous insects depend on a mixture of compounds in their location of host plants (Bruce and Pickett 2011; Visser 1986). The mixture of cucumber and tomato are more attractive to melon flies (Baig et al. 2023; Njuguna et al. 2018; Siderhurst and Jang 2010). Thus, methyl isovalerate, methyl myristate, 1-octadecene, and heptanal could be mixed in certain proportions for further experiments, laying the foundation for the selection of attractants and repellents.

In conclusion, we found that several volatile compounds could be used as behavioral regulators in melon flies. Field experiments will be conducted on volatile mixtures to develop a synthetic bait for melon flies, which laid the foundation for monitoring and effectively controlling *Z. cucurbitae* in an environmentally-friendly manner.

## Supplementary Information

Below is the link to the electronic supplementary material.

Supplementary file1 (DOCX 31 KB)

## Data Availability

Availability of Data and Material All data are available in this paper
