# Supplementary material for: Electroantennographic and Behavioral Responses of the Melon fly, Zeugodacus cucurbitae (Coquillett), to Volatile Compounds of Ridge Gourd, Luffa acutangular L
Source: J Chem Ecol. 2024 Feb 19;50(12):1036–45. doi: 10.1007/s10886-024-01474-1 (PMC11717787; doi:10.1007/s10886-024-01474-1)
Supplement: Supplementary file 1 — Supplementary file1 (DOCX 31 KB) [file 10886_2024_1474_MOESM1_ESM.docx]

# Supplementary Material

# Electroantennographic and Behavioral Responses of the Melon fly, *Zeugodacus* *cucurbitae* (Coquillett), to Volatile Compounds of Ridge Gourd, *Luffa acutangular* L.

### **Jing jingWang^1,2^, Chao Ma^1,2^, Zhen yaTian^2,3^, Yong ping Zhou^2,3^, Jin fang Yang^1,2^,** **XuyuanGao^2,3^, Hong song Chen^2,3^, Wei hua Ma^4^ and Zhong shi Zhou^1,2^***

^1^ State Key Laboratory for Biology of Plant Diseases and Insect Pests, Institute of Plant Protection, Chinese Academy of Agricultural Sciences, Beijing, 100193, China.

^2^ National Nanfan Research Institute, Chinese Academy of Agricultural Sciences, Sanya, 572019, China,

^3^ Guangxi Key Laboratory for Biology of Crop Diseases and Insect Pests, Institute of Plant Protection, Guangxi Academy of Agricultural Sciences, Nanning, 530007, China.

^4^ Hubei Insect Resources Utilization and Sustainable Pest Management Key Laboratory, College of Plant Science and Technology, Huazhong Agricultural University, Wuhan, 430070, China.

Correspondence: email: [zhouzhongshi@caas.cn](mailto:zhongshizhou@yahoo.com).

**Table S1**. Analysis of data of EAG responses of *Zeugodacus* *cucurbitae* to synthetic compounds from *Luffa acutangular*

| Compound | *F* | *P* | *df* |
| --- | --- | --- | --- |
| methyl stearate | 3.22 | 0.075 | 1 |
| methyl myristate | 3.168 | 0.067 | 1 |
| p-xylene | 16.857 | <0.001 | 1 |
| phytane | 4.746 | 0.051 | 1 |
| alpha-pinene | 17.221 | <0.001 | 1 |
| methyl isovalerate | 4.364 | 0.056 | 1 |
| 1-octadecene | 7.109 | 0.009 | 1 |

**Table S2**. Analysis of data of EAG dose responses of *Zeugodacus* *cucurbitae* to synthetic compounds from *Luffa acutangular*

| Compound | female | | | male | | |
| --- | --- | --- | --- | --- | --- | --- |
|  | *F* | *P* | *df* | *F* | *P* | *df* |
| methyl stearate | 30.298 | <0.001 | 4 | 30.664 | <0.001 | 4 |
| methyl myristate | 14.171 | <0.001 | 4 | 10.606 | <0.001 | 4 |
| p-xylene | 41.603 | <0.001 | 4 | 48.721 | <0.001 | 4 |
| phytane | 16.629 | <0.001 | 4 | 10.887 | <0.001 | 4 |
| alpha-pinene | 12.141 | 0.009 | 4 | 3.361 | 0.016 | 4 |
| methyl isovalerate | 7.902 | <0.001 | 4 | 3.507 | 0.013 | 4 |
| 1-octadecene | 14.473 | <0.001 | 4 | 11.073 | 0.009 | 4 |

**Table S3.** Analysis of data of behavioral responses of *Zeugodacus* *cucurbitae* to 100 µg/µL of synthetic compounds from *Luffa acutangular*

| Compound | female | | | male | | |
| --- | --- | --- | --- | --- | --- | --- |
|  | *χ^2^* | *P* | *df* | *χ^2^* | *P* | *df* |
| methyl stearate | 16.396 | <0.001 | 1 | 0.053 | 0.818 | 1 |
| methyl myristate | 0.157 | 0.843 | 1 | 0.717 | 0.498 | 1 |
| p-xylene | 2.686 | 0.144 | 1 | 1.228 | 0.376 | 1 |
| phytane | 1.125 | 0.377 | 1 | 1.697 | 0.264 | 1 |
| alpha-pinene | 0.13 | 0.857 | 1 | 0.416 | 0.629 | 1 |
| methyl isovalerate | 43.781 | <0.001 | 1 | 12.645 | ＜0.001 | 1 |
| 1-octadecene | 1.228 | 0.376 | 1 | 3.03 | 0.126 | 1 |

**Table S4.** Analysis of data of behavioral responses of *Zeugodacus* *cucurbitae* to 10 µg/µL of synthetic compounds from *Luffa acutangular*

| Compound | female | | | male | | |
| --- | --- | --- | --- | --- | --- | --- |
|  | *χ^2^* | *P* | *df* | *χ^2^* | *P* | *df* |
| methyl stearate | 3.854 | 0.073 | 1 | 1.705 | 0.276 | 1 |
| methyl myristate | 16.629 | ＜0.001 | 1 | 12.500 | ＜0.001 | 1 |
| *p*-xylene | 2.381 | 0.176 | 1 | 22.588 | ＜0.001 | 1 |
| phytane | 1.228 | 0.376 | 1 | 0 | 1 | 1 |
| alpha-pinene | 3.947 | 0.068 | 1 | 1.705 | 0.276 | 1 |
| methyl isovalerate | 12.980 | ＜0.001 | 1 | 20.275 | ＜0.001 | 1 |
| 1-octadecene | 0.385 | 0.680 | 1 | 2 | 0.238 | 1 |

**Table S5.** Analysis of data of behavioral responses of *Zeugodacus* *cucurbitae* to 10 µg/µL of synthetic compounds from *Luffa acutangular*

| Name | female | | | male | | | |
| --- | --- | --- | --- | --- | --- | --- | --- |
|  | *χ^2^* | *P* | *df* | | *χ^2^* | *P* | *df* |
| heptanal | 1.330 | 0.357 | 1 | | 0.307 | 0.783 | 1 |
| methyl stearate | 0.327 | 0.704 | 1 | | 0.344 | 0.696 | 1 |
| 1-decanol | 6.666 | 0.01 | 1 | | 0.13 | 0.718 | 1 |
| methyl myristate | 15.672 | ＜0.001 | 1 | | 13.530 | ＜0.001 | 1 |
| citronellol | 1.087 | 0.435 | 1 | | 2 | 0.238 | 1 |
| p-xylene | 10.965 | 0.01 | 1 | | 2.446 | 0.191 | 1 |
| phytane | 0.189 | 0.828 | 1 | | 2.057 | 0.251 | 1 |
| alpha-pinene | 3.125 | 0.111 | 1 | | 0.038 | 0.845 | 1 |
| methyl isovalerate | 23.629 | ＜0.001 | 1 | | 27.125 | ＜0.001 | 1 |
| 1-octadecene | 0.442 | 0.506 | 1 | | 0.907 | 0.446 | 1 |

**Table S6.** Analysis of data of behavioral responses of *Zeugodacus* *cucurbitae* to 0.1 µg/µL of synthetic compounds from *Luffa acutangular*

| Name | female | | | male | | | |
| --- | --- | --- | --- | --- | --- | --- | --- |
|  | *χ^2^* | *P* | *df* | | *χ^2^* | *P* | *df* |
| heptanal | 4.289 | 0.063 | 1 | | 0.385 | 0.68 | 1 |
| methyl stearate | 9.765 | 0.002 | 1 | | 2.940 | 0.086 | 1 |
| 1-decanol | 9.758 | 0.002 | 1 | | 4.013 | 0.045 | 1 |
| methyl myristate | 13.235 | ＜0.001 | 1 | | 0.026 | 0.81 | 1 |
| citronellol | 2.400 | 0.168 | 1 | | 4.190 | 0.062 | 1 |
| *p*-xylene | 2.58 | 0.153 | 1 | | 0.707 | 0.529 | 1 |
| phytane | 0.829 | 0.467 | 1 | | 3.703 | 0.086 | 1 |
| alpha-pinene | 0.189 | 0.828 | 1 | | 1.405 | 0.31 | 1 |
| methyl isovalerate | 26.611 | ＜0.001 | 1 | | 17.887 | ＜0.001 | 1 |
| 1-octadecene | 7.219 | 0.007 | 1 | | 2.381 | 0.176 | 1 |

**Table S7.** Analysis of data of behavioral responses of *Zeugodacus* *cucurbitae* to 0.01 µg/µL of synthetic compounds from *Luffa acutangular*

| Name | female | | | male | | |
| --- | --- | --- | --- | --- | --- | --- |
|  | *χ^2^* | *P* | *df* | *χ^2^* | *P* | *df* |
| heptanal | 0.829 | 0.467 | 1 | 0.707 | 0.529 | 1 |
| methyl stearate | 2.485 | 0.161 | 1 | 0.287 | 0.721 | 1 |
| 1-decanol | 2.607 | 0.165 | 1 | 3.556 | 0.097 | 1 |
| methyl myristate | 13.235 | 0.026 | 1 | 0.038 | 0.845 | 1 |
| citronellol | 0.482 | 0.603 | 1 | 0.5 | 0.596 | 1 |
| p-xylene | 2.24 | 0.199 | 1 | 0 | 1 | 1 |
| phytane | 0.033 | 0.856 | 1 | 11.607 | 0.001 | 1 |
| alpha-pinene | 0.829 | 0.467 | 1 | 0.312 | 0.71 | 1 |
| methyl isovalerate | 19.444 | ＜0.001 | 1 | 25.293 | ＜0.001 | 1 |
| 1-octadecene | 21.597 | ＜0.001 | 1 | 0.125 | 0.724 | 1 |
